# Experimental measurements of Xe and Kr releases from UO_2_ and determination of their migration mechanisms – Release rate data

**DOI:** 10.1016/j.dib.2021.107645

**Published:** 2021-11-26

**Authors:** M. Gérardin, E. Gilabert, D. Horlait, M-F. Barthe, G. Carlot

**Affiliations:** a CEA, DEN, DEC, F-13108 Saint Paul lez Durance Cedex, France; b Université de Lorraine, GeoRessources, CNRS, F54000 Nancy, France; c Université de Bordeaux, CNRS, CENBG-IN2P3, F-33170 Gradignan, France; d CEMHTI/CNRS, Université d'Orléans, 3A rue de La Férollerie, 45071 Orléans CEDEX 2, France

**Keywords:** Uranium dioxide, Xenon and krypton release, Diffusion, Radiation induced defects

## Abstract

We present the raw data obtained from release rate at 1300°C of Xe and Kr implanted in UO_2_, related to [1]. We performed different sample preparation (polishing treatment) on polycrystalline and monocrystalline UO_2_. Ion implantation were performed at various fluences between 9.5 × 10^10^ to 5 × 10^14^ i/cm^2^ in UO_2_ samples. Release rate of Xe and Kr are obtained at 1300°C under vacuum from desorption experiments performed on the PIAGARA plateform at the CENBG (Centre d'Etudes Nucléaires de Bordeaux-Gradignan). Since we made a variety of samples depending on multiple parameters (sample type, sample preparation, ion implantation type and fluence), these data represent a serious amount of work that could be saved for the scientific community that might use them for other purposes such as burst modelling.

## Specifications Table

**Table:** 

Subject	Material Physics
Specific subject area	Understanding the migration mechanisms of xenon and krypton in UO_2_
Type of data	Table
How the data were acquired	Thermal Desorption Spectrometry measurements performed on the PIAGARA plateform at the CENBG (Centre d'Etudes Nucléaires de Bordeaux-Gradignan). Samples are under ultra-high vacuum at 1300°C.
Data format	Raw
Parameters for data collection	Under vacuum (10^−9^ mbar) 1300°C
Description of data collection	Every 20 minutes, a fraction of the gas released from the sample is analysed in the mass spectrometer. 20 minutes is the minimum time span required to expand gas samplings, to do the measurements in the mass spectrometer and to evacuate analyzed gas by pumping in-between measurements. After the burst release, samplings were more spaced out. Every point in the .dat raw data corresponds to a sampling.
Data source location	• Institution: Centre Nucléaire de Bordeaux Gradignan (CENBG) • City/Town/Region: Bordeaux • Country: France
Data accessibility	Repository name: Mendeley Data Data identification number: DOI:10.17632/gj565g85fb.1 Direct URL to data: https://data.mendeley.com/datasets/gj565g85fb/1
Related research article	[1] M. Gerardin, E. Gilabert, D. Horlait, M.-F. Barthe, and G. Carlot, “Experimental study of the diffusion of Xe and Kr implanted at low concentrations in UO2 and determination of their trapping mechanisms,” J. Nucl. Mater., vol. 556, p. 153174, Dec. 2021, doi:10.1016/j.jnucmat.2021.153174.

## Value of the Data

• Understanding of rare gases migration in UO2 remains a challenge since it depends on multiple parameters. Working on radioactive samples implies multiple challenges related to preparation, transport and destructive analysis. The implantation at such low concentrations was another challenge. Considering the long and challenging process to obtain them, these new data on the release rates of xenon and krypton implanted in UO2 are valuable for promising discussions on the parameters affecting the diffusion.
• These data on rare gases diffusion in UO2 would benefit researchers working on gases behavior in fuel simulations, along with experimental researchers
• The scientific community interested in diffusion processes in nuclear materials could use these data for modelling purposes

## 1 Data Description

The reader will find a pdf file presenting sample description and characteristics. The release rates at 1300°C under vacuum of each sample are presented in separated .dat files. Each .dat file has 4 columns: (1) Time (min) corresponding to the time of the gas sampling; (2) Temperature (°C); (3) Fractional release (Fr) (see description below) and (4) Fractional release error (Fre) related only to mass spectrometry measurements.

## 2 Experimental Design, Materials and Methods

Desorption experiments at 1300°C were performed on the PIAGARA platform at the CENBG (Centre d'Etudes Nucléaires de Bordeaux-Gradignan). More details about the setup can be found in these references [2,3]. This experimental device is made of (1) the heating chamber comprising the sample placed in a platinum crucible and a set of valves and various calibrated volumes that allow to take gas samplings, (2) several chemical traps (for the purification of gas samplings), (3) the calibration setup: a 82Kr or 129Xe calibrated monoisotopic reference gas with a specific precisely known concentration and (4) the mass spectrometer measuring the isotopic ratio between Kr or Xe released from the sample and from the reference gas addition.

The sample is placed in the furnace at 1300°C at t=0. After 10 min annealing (t_1_), a first sampling is performed. The sampled gas is purified and analysed in the mass spectrometer. The intensity (pA) of 83Kr or 131Xe measured by the spectrometer is converted to a number of atoms via a quantitative calibration of the spectrometer performed using calibrated amount of 82Kr or 129Xe. The fractional release (Fr) at a time t is then calculated using the number of atoms released, divided by the number of atoms implanted in the sample (i.e. the implantation fluence). Then, the Fr value ranges between 0 and 1 (Fr=1 corresponding to the total release of the gas implanted in the sample). After the measurement, the chamber of the mass spectrometer (as well as the volumes used for the sampling) is pumped, but the chamber of the furnace with the sample is not. After 20 min (t_2_), another sampling is performed and analysed similarly to the first one. Since the furnace chamber (containing the heated sample) is not pumped, the Fr(t_2_) value is either the same (no gas release during t_1_ and t_2_) or higher (gas released during t_1_ and t_2_) than Fr(t_1_). That is why the Fr value is either increasing or stable with the time. After 7 or 8 hours annealing comprising around 10 sampling and analysis in the mass spectrometer, all the extraction line is pumped and the furnace chamber is isolated so to remove the sample from the Pt crucible and to place another sample in the same crucible. The crucible is placed in the cold part of the furnace and the chamber is pumped during the night so that the pressure is sufficiently low the next morning for further heating experiment.

**Ethics Statements**

## CRediT Author Statement

**Eric Gilabert:** Experimental work, Investigation, Model development; **Denis Horlait:** Experimental work, Investigation; **Gaelle Carlot and Marie-France Barthe:** Supervision; **Marie Gerardin:** Experimental work, sample preparation, Investigation, Writing.

## Declaration of Competing Interest

The authors declare that they have no known competing financial interests or personal relationships that could have appeared to influence the work reported in this paper.
